# Women's Autonomy in Health Care Decision-Making in Somalia: Evidence from a Multinomial Logistic Regression Analysis of the 2020 National Survey

**DOI:** 10.1186/s12905-026-04619-4

**Published:** 2026-07-04

**Authors:** Hamda Jama Yousuf, Abdiasis Aden Omer, Suhur A. Ahmed

**Affiliations:** 1 Ogaansho Research and Consultancy Center, Burao, Somaliland; 2 Ogaansho Research and Consultancy Center, Burao, Somaliland

**Keywords:** Women’s autonomy, Maternal health services, Somalia

## Abstract

**Background:**

Women’s autonomy in healthcare decision-making is a key determinant of maternal and reproductive health outcomes in Somalia. This study examined the level of women’s autonomy and its associated factors using nationally representative data.

**Methods:**

A cross-sectional study was conducted using data from the 2020 Somalia Demographic and Health Survey, including 32,272 women aged 15–49 years. Data were analyzed using STATA version 17, accounting for sampling weights. Multinomial logistic regression was used, and results were reported as Relative Risk Ratios (RRRs) with 95% Confidence Intervals (CIs).

**Results:**

Overall, 15.3% of women made healthcare decisions independently, 32.9% made decisions jointly, and 51.8% had decisions made by others. Older women aged 45–49 years were more likely to make independent decisions compared to those aged 15–19 (RRR = 1.93; 95% CI: 1.42–2.62). Urban residence was associated with higher autonomy (RRR = 1.17; 95% CI: 1.07–1.28). Women in the highest wealth quintile had a higher likelihood of independent decision-making compared to the lowest quintile (RRR = 2.68; 95% CI: 2.36–3.03). Higher maternal education was also significantly associated with increased autonomy.

**Conclusion:**

Women’s autonomy in healthcare decision-making in Somalia remains limited and is associated with socio-demographic and economic factors. Interventions focusing on improving women’s education, economic empowerment, and access to healthcare services may enhance autonomy and improve health outcomes.

## Background study

Autonomy is seen as crucial for decision-making in several healthcare contexts, including healthcare seeking, utilization, and the selection of treatment alternatives. Evidence indicates that women in developing or low-income nations often possess restricted autonomy and authority over their health choices [1]. Research on women’s autonomy in developing countries sought to characterize the link between women’s autonomy and healthcare decision-making, as well as to uncover sociodemographic variables influencing women’s autonomy and healthcare decision-making [1]. A key factor in determining the health of mothers and children is women’s autonomy, which is the capacity to make choices on their own in a variety of areas of life, including healthcare. Women’s autonomy is essential for managing sexual health, including the prevention and treatment of STIs, using contraceptives, and accessing reproductive healthcare. When women are empowered to make healthcare decisions on their own, they are better able to manage their own and their children’s health [2]. However, in many poor nations, women’s autonomy is often curtailed. Traditionally, men are the key decision-makers in households, limiting women’s capacity to make health-related choices. This lack of autonomy directly affects maternal health outcomes. Women with limited decision-making power are less likely to access essential services like antenatal care, skilled delivery, and postnatal care, which are vital for reducing maternal mortality. Furthermore, insufficient use of maternal health services significantly increases the risk of complications and maternal death [2].

Globally, maternal mortality remains a pressing issue, particularly in low- and middle-income countries (LMICs), where gender inequality and limited women autonomous healthcare seeking persist, In 2020, approximately 287 000 maternal deaths occurred worldwide, with 800 women dying each day from preventable pregnancy and childbirth complications [2].

Sub-Saharan Africa bears a disproportionate share of these deaths, where a woman’s lifetime risk of dying from maternal causes is 1 in 37, compared to 1 in 4800 in high-income countries [2]. Although most maternal deaths are preventable, the most recent available data indicate that the maternal mortality rate in Africa is among the highest worldwide, According to the 2015 World Health Organization (WHO) report, 303,000 women worldwide died during pregnancy or after childbirth, and most of these deaths occurred in sub-Saharan Africa, In Ethiopia, the maternal mortality ratio remains high, estimated between 267 and 401 per 100,000 live births depending on the data source and year. Although significantly reducing maternal mortality in sub-Saharan African countries appears difficult, skilled care before, during, and after childbirth is highly crucial for saving the lives of women and newborn infants, Maternal healthcare service utilization is strongly recommended as the most critical strategy for reducing maternal mortality rates in low-income countries [3]. Studies have shown that in Ethiopia, maternal healthcare service utilization is below acceptable standards, Only 19% of women made four or more antenatal care (ANC) visits during their pregnancy, only 11% of pregnant women had health facility delivery (HFD), and only 9.7% of women received postnatal care (PNC) in the first 2 days after delivery, Reasons for the low maternal healthcare service utilization include the lack of transportation, a long distance to a health facility, cultural beliefs inculcating a preference for home births, fear of health facility treatments, and lack of awareness regarding the importance of skilled delivery [3].

In Somalia, maternal mortality remains high, with limited evidence on the factors influencing women’s decision-making autonomy. Previous studies in other countries suggest that education, wealth, and urban residence are associated with higher autonomy; however, these findings cannot be directly generalized to Somalia due to unique cultural, social, and economic contexts. Therefore, this study aims to examine the determinants of women’s autonomy in healthcare decision-making using the nationally representative 2020 Somalia Demographic and Health Survey (DHS) dataset. Understanding these factors is essential to inform policies promoting gender equity and improving maternal health outcomes in Somalia. Understanding these factors is crucial for informing policies that promote gender equity and improve maternal health outcomes, particularly in disadvantaged areas. Enhancing women’s autonomy aligns with global health objectives, including Sustainable Development Goal (SDG) 3, which promotes healthy lives and well-being for all, and SDG 5, which focuses on achieving gender equality and empowering women and girls. This study seeks to fill the knowledge gap by assessing the level of women’s autonomy and identifying sociodemographic, economic, and reproductive factors that influence decision-making in Somalia.

## Methods and material

The first nationally representative survey conducted by the Somalia National Health Organization was the 2020 Somalia Demographic and Health Survey (SDHS). Bureau of Statistics, January 2018 to February 2019. Somalia, which is estimated to measure 637,657 km2 and located in the Horn of Africa, is mostly composed of plateaus, plains, and mountains. Its coastline, which stretches over 3333 km along the Gulf of Aden to the north and the Indian Ocean to the east and south, is the longest in Africa. Somalia has borders with Djibouti to the northwest, Ethiopia to the west, and Kenya to the southwest [4]. 

The 2020 Somalia Demographic and Health Survey (SDHS) employed a three-stage stratified cluster sampling design to ensure national representativeness. The country was stratified into urban, rural, and nomadic areas across administrative regions. In the first stage, Enumeration Areas (EAs) were selected using probability proportional to size. In the second stage, households were systematically selected from each EA based on updated household listings. In the third stage, eligible women aged 15–49 years were interviewed. Due to security constraints, some regions such as Lower Shabelle, Middle Juba, and parts of Bay were excluded from the sampling frame. These exclusions may affect full national representation, but are consistent with DHS survey procedures in fragile settings [5].

### Sample size

The final analytic sample included 32,272 women with complete information on autonomy and covariates.

### Study variables

#### Outcome variable

The outcome of this study was women’s autonomy in healthcare decision-making, which reflects the ability of women to make decisions regarding their own health. Autonomy was assessed by asking respondents who usually makes decisions about their healthcare. Responses were categorized into three groups: (1) Independently, when the woman makes decisions alone; (2) Together, when decisions are made jointly by the woman and her husband; and (3) With others, when decisions are made by other people without the woman’s involvement. Women who made decisions independently were considered fully autonomous. For the purposes of regression analysis, the “With others” category was used as the reference group, representing low autonomy. This categorization allows for a clear understanding of different levels of decision-making power among women in the Somali context.

#### Independent variables

The 15 explanatory factors that made up the independent variables were chosen based on their theoretical significance and previous research findings. Included Age of the women, region, type place of residence, sex of household head age of household head, family size, wealth index, maternal employment, husband’s employment, maternal education, husband’s education, parity, age at first marriage, distance from health facility, media exposure.

### Data analysis

Data from the 2020 Somalia Demographic and Health Survey (SDHS) were cleaned, coded, and analyzed using STATA version 17. There are several phases to the data analyzing process. First, the sociodemographic, economic, reproductive, and household characteristics of the research participants were compiled using descriptive statistics. Sampling weights provided by the 2020 Somalia Demographic and Health Survey (SDHS) were applied to account for the complex survey design and to ensure representativeness at the national level. The distribution of women’s autonomy in making healthcare decisions and other categorical factors was described using frequencies and percentages.

Second, the relationship between each independent variable and women’s autonomy in making healthcare decisions was investigated using bivariate analysis. To find statistically significant correlations, the Pearson chi-square (χ²) test was used. Candidates for inclusion in the multivariable analysis were variables whose bivariate p-value was less than 0.25.

Lastly, multinomial logistic regression was used to examine the factors that influence women’s autonomy in making decisions about their health care. Because logistic regression is suitable for analyzing the connection between a categorical result variable and one or more categorical or continuous predictor variables, this approach was used. When the dependent variable has three or more nominal categories, multinomial logistic regression is especially appropriate. Women’s autonomy in making decisions about their health care was categorized in this research as (1) making decisions on their own (2), making decisions with her Husband, and (3) having others make decisions. in the multivariable model was set at *p* < 0.05. Multicollinearity among independent variables was checked using variance inflation factors (VIF), and all variables included in the model had VIF values below 10, indicating no significant multicollinearity.

## Result

As shown in Table 1, the majority of respondents were in the middle and later reproductive ages, with women aged 30–34 and 35–39 years accounting for 22.35% and 23.29%, respectively, while only 1.81% were aged 15–19 years. Regional variation was observed, with Banadir, Sool, and Sanaag contributing the largest shares of the sample (9.63%, 9.17%, and 8.96%, respectively), whereas Bay accounted for the smallest proportion (1.79%). Regarding place of residence, 38.42% of women lived in urban areas, 34.45% in nomadic settings, and 27.13% in rural areas. Socioeconomic status was generally low, with 24.0% of women in the lowest wealth quintile and only 16.45% in the highest quintile. Husbands’ employment and education levels were low, with 56.24% unemployed and 88.47% having no formal education. Maternal employment was rare (1.13%), and most women (87.06%) had no formal education. Most households were male-headed (66.33%), and a large proportion had four or more members (77.25%). High fertility was evident, as 69.3% of women were grand multipara, and early marriage was common, with 73.45% marrying before age 20. Access to health services was a challenge for over one-third of respondents (35.38%), and media exposure was limited, with only 27.36% of women reporting access.

**Table 1:** Sociodemographic Characteristics Among Reproductive Age Women in Somalia

Variable	Category	Frequency	Percentage
Age	15–19	585	1.81
	20–24	3272	10.14
	25–29	7146	22.14
	30–34	7213	22.35
	35–39	7517	23.29
	40–44	4330	13.42
	45–49	2209	6.84
Region	Awdal	1570	4.86
	Woqooyi Galbeed	2410	7.47
	Togdheer	2565	7.95
	Sool	2958	9.17
	Sanaag	2893	8.96
	Bari	2013	6.24
	Nugaal	1822	5.65
	Mudug	1776	5.50
	Galgaduud	1675	5.19
	Hiraan	1448	4.49
	Middle Shabelle	1661	5.15
	Banadir	3107	9.63
	Bay	579	1.79
	Bakool	2054	6.36
	Gedo	1919	5.95
	Lower Juba	1822	5.65
Type of place of residence	Rural	8756	27.13
	Urban	12,399	38.42
	Nomadic	11,117	34.45
Wealth index	Lowest	7745	24.00
	Second	7352	22.78
	Middle	6049	18.74
	Fourth	5818	18.03
	Highest	5308	16.45
Husband employment	Yes	13,765	43.76
	No	17,691	56.24
Husband’s educational status	No	28,551	88.47
	Yes	3721	11.53
Maternal employment	Yes	366	1.13
	No	31,906	98.87
Maternal educational status	No Education	28,095	87.06
	Primary	3214	9.96
	Secondary	796	2.47
	Higher	167	0.52
Sex of household head	Male	21,405	66.33
	Female	10,867	33.67
Age of household head	15–24 years	6225	19.29
	25–34 years	11,259	34.89
	35 and above	14,788	45.82
Family size	Less than four members	7343	22.75
	Four members or more	24,929	77.25
Parity	Primipara	816	2.53
	Multipara	9093	28.18
	grand multipara	22,363	69.30
Age at First Marriage	< 20	23,104	73.45
	20–24	6760	21.49
	25–30	1489	4.73
	30 and above	103	0.33
distance to health facility	Not a big problem	20,853	64.62
	Big problem	11,419	35.38
media exposure	No	22,849	72.64
	Yes	8607	27.36

**Fig. 1:**
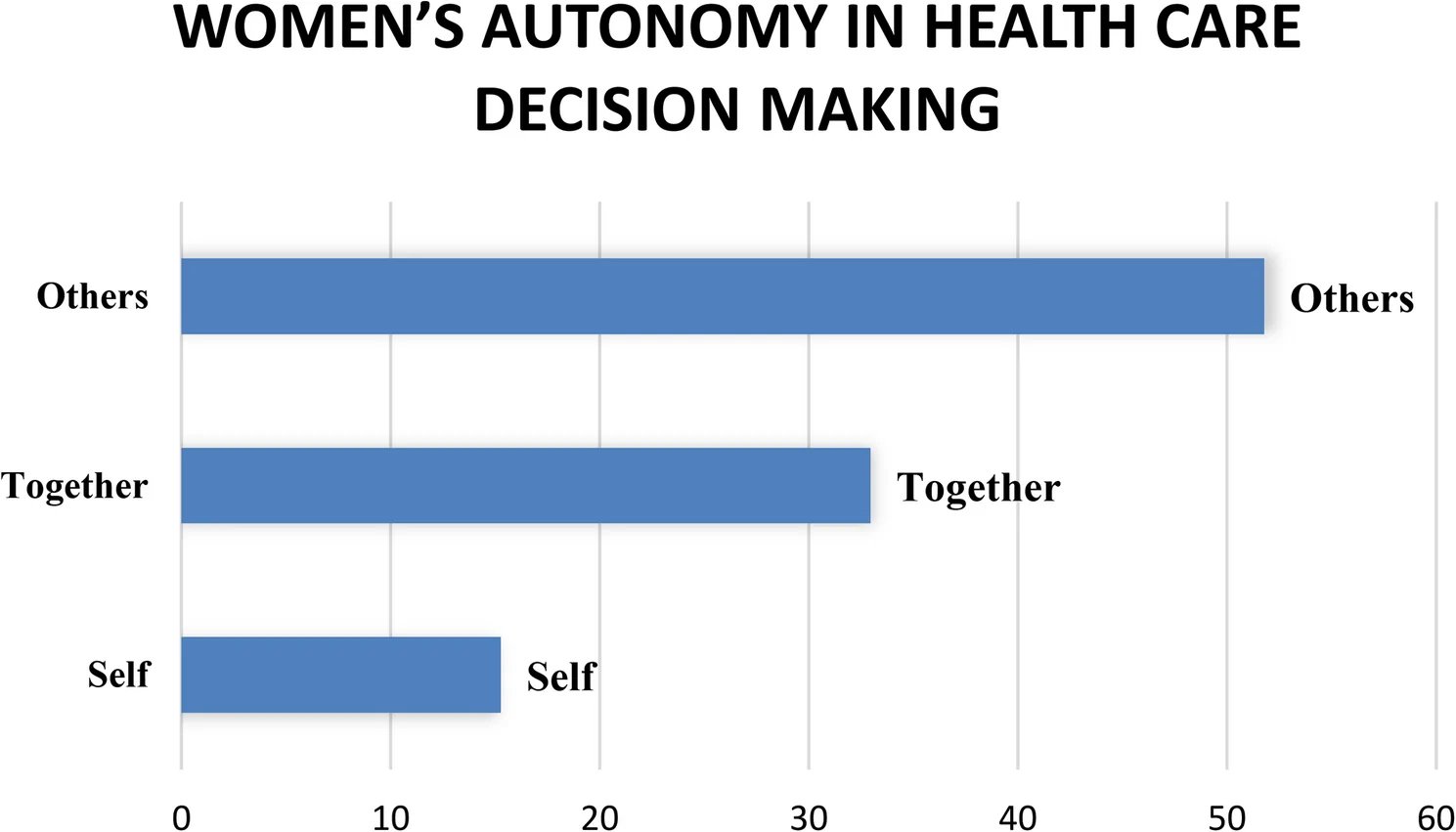
shows the autonomy of women in Somalia’s health care decision-making process. Only 15.3% of the women made choices on their own, while 32.9% made decisions together, and more than half (51.8%) said that others made decisions about their health care

Figure 1. Distribution of women’s autonomy in healthcare decision-making among reproductive-age women in Somalia (*n* = 32,272). “Self” refers to women making decisions independently, “Together” indicates joint decision-making with a partner, and “Others” refers to decisions made by other individuals without the woman’s involvement.

Table 2 presents the bivariate analysis examining the associations between various independent variables including socio-demographic, economic, household, and reproductive characteristics and women’s autonomy in health care decision-making among reproductive-age women in Somalia. The results show that women’s age was significantly associated with autonomy, with decision-making increasing as age increased (χ² = 290.90, *p* < 0.05). Educational attainment was also significantly associated, as women with primary, secondary, or higher education were more likely to make independent health care decisions compared to those with no formal education (χ² = 197.91, *p* < 0.05).

**Table 2:** Bivariate Analysis on Women’s Autonomy in Health Care Among Reproductive-Age Women in Somalia

Variable	Category	Women’s autonomy in health care decision making			X2 (*p*-value)
		Self	Together	Others	
Age	15–19	84(14.51)	154(26.60)	341(58.89)	290.90 *
	20–24	384(11.93)	1006(31.26)	1828(56.81)	
	25–29	913(13.15)	2149(30.95)	3882(55.90)	
	30–34	1073(15.23)	2404(34.13)	3566(50.63)	
	35–39	1076(14.62)	2501(33.98)	3784(51.41)	
	40–44	772(18.51)	1377(33.02)	2021(48.47)	
	45–49	502(23.45)	745(34.80)	894(41.76)	
Region	Awdal	189(12.47)	563(37.14)	764(50.40)	1544.44 *
	Woqooyi Galbeed	424(18.05)	900(38.31)	1025(43.64)	
	Togdheer	543(21.59)	759(30.18)	1213(48.23)	
	Sool	417(14.33)	1179(40.52)	1314(45.15)	
	Sanaag	475(17.06)	819(29.41)	1491(53.54)	
	Bari	247(12.44)	793(39.95)	945(47.61)	
	Nugaal	344(19.36)	519(29.21)	914(51.44)	
	Mudug	271(16.06)	559(33.14)	857(50.80)	
	Galgaduud	245(15.01)	640(39.22)	747(45.77)	
	Hiraan	196(13.90)	528(37.45)	686(48.65)	
	Middle Shabelle	262(16.00)	530(32.36)	846(51.65)	
	Banadir	646(21.31)	1089(35.92)	1297(42.78)	
	Bay	136(23.49)	234(40.41)	209(36.10)	
	Bakool	177(8.66)	564(27.58)	1304(63.77)	
	Gedo	81(4.39)	268(14.52)	1497(81.09)	
	Lower Juba	151(8.63)	392(22.40)	1207(68.97)	
Type of place of residence	Rural	1382(16.23)	3055(35.87)	4080(47.90)	868.34 *
	Urban	2368(19.61)	4258(35.25)	5452(45.14)	
	Nomadic	1054(9.70)	3023(27.83)	6784(62.46)	
Wealth index	Lowest	1004(13.31)	2451(32.49)	4089(54.20)	687.21 *
	Second	835(11.67)	1925(26.91)	4394(61.42)	
	Middle	1003(17.10)	1844(31.45)	3017(51.45)	
	Fourth	1067(18.75)	1880(33.04)	2743(48.21)	
	Highest	895(17.20)	2236(42.97)	2073(39.83)	
Husband employment	Yes	1790(13.00)	4694(34.10)	7281(52.90)	98.91 *
	No	3014(17.04)	5642(31.89)	9035(51.07)	
Husband’s educational status	No	4282(15.39)	8944(32.14)	14,601(52.47)	56.691 *
	Yes	522(14.38)	1392(38.36)	1715(47.26)	
Maternal employment	Yes	105(29.75)	127(35.98)	121(34.28)	71.32 *
	No	4699(15.11)	10,209(32.82)	16,195(52.07)	
Maternal educational status	No Education	4095(14.97)	8676(31.73)	14,575(53.30)	197.91 *
	Primary	575(18.21)	1234(39.09)	1348(42.70)	
	Secondary	105(13.34)	343(43.58)	339(43.07)	
	Higher	29(17.47)	83(50.00)	54(32.53)	
Sex of household head	Male	3152(15.17)	6692(32.21)	10,934(52.62)	15.03 *
	Female	1652(15.47)	3644(34.13)	5382(50.40)	
Age of household head	15–24 years	1153(19.15)	2157(35.82)	2712(45.03)	742.64*
	25–34 years	2022(18.41)	4011(36.51)	4953(45.08)	
	35 and above	1629(11.27)	4168(28.85)	8651(59.88)	
Family size	Less than four members	1014(14.10)	2320(32.27)	3855(53.62)	14.82 *
	Four members or more	3790(15.62)	8016(33.03)	12,461(51.35)	
Parity	Primipara	123(15.38)	254(31.75)	423(52.88)	13.62 *
	Multipara	1328(14.96)	2806(31.61)	4744(53.44)	
	grand multipara	3353(15.40)	7276(33.41)	11,149(51.19)	
Age at First Marriage	< 20	3243(14.04)	7483(32.39)	12,378(53.58)	157.61 *
	20–24	1244(18.40)	2272(33.61)	3244(47.99)	
	25–30	290(19.48)	553(37.14)	646(43.38)	
	30 and above	27(26.21)	28(27.18)	48(46.60)	
Distance to health facility	Not a big problem	3113(15.31)	6144(30.21)	11,078(54.48)	197.92 *
	Big problem	1691(15.21)	4192(37.69)	5238(47.10)	
Media exposure	No	3229(14.13)	7318(32.03)	12,302(53.84)	151.19 *
	Yes	1575(18.30)	3018(35.06)	4014(46.64)	

*p-value less than < 0.05

Regional differences were evident (χ² = 1544.44, *p* < 0.05), with women in Banadir, Bay, and Togdheer demonstrating higher autonomy than those in Gedo and Lower Juba. Women residing in urban areas were more likely to participate in decision-making than those in rural or nomadic settings (χ² = 868.34, *p* < 0.05). Economic factors, including wealth index, maternal employment, and husband’s education and employment, were significantly associated with autonomy. Women from higher wealth quintiles, employed women, and those whose husbands were educated exhibited greater participation in health care decisions (χ² = 687.21, 71.32, 56.69, respectively; all *p* < 0.05).

Reproductive factors, including parity (χ² = 13.62, *p* < 0.05), age at first marriage (χ² = 157.61, *p* < 0.05), and perceived distance to health facilities (χ² = 197.92, *p* < 0.05), were also significantly linked to autonomy. Exposure to media increased the likelihood of women making independent or joint decisions (χ² = 151.19, *p* < 0.05). In contrast, household size and sex of the household head were not significantly associated with autonomy. Overall, these findings highlight that age, education, region, wealth, employment, reproductive factors, and access to information and health services play important roles in shaping women’s autonomy in health care in Somalia.

The multivariable multinomial logistic regression analysis in Table 3 identifies factors independently associated with women’s autonomy in health-care decision-making among reproductive-age women in Somalia. Age was positively associated with joint decision-making: women aged 30–34 (RRR = 1.403, 95% CI: 1.12–1.757, *p* < 0.001), 35–39 (RRR = 1.352, 95% CI: 1.077–1.699, *p* < 0.001), 40–44 (RRR = 1.43, 95% CI: 1.132–1.808, *p* < 0.001), and 45–49 (RRR = 1.611, 95% CI: 1.259–2.062, *p* < 0.001) were more likely to make healthcare decisions together with their partners compared to women aged 15–19. Regional variations were observed, with women in Woqooyi Galbeed (RRR = 1.146, 95% CI: 0.99–1.326, *p* < 0.05), Sool (RRR = 1.159, 95% CI: 1.006–1.334, *p* < 0.01), Bari (RRR = 1.173, 95% CI: 1.009–1.364, *p* < 0.01), and Galgaduud (RRR = 1.231, 95% CI: 1.051–1.441, *p* < 0.01) showing higher likelihood of joint decision-making, whereas women in Togdheer, Sanaag, Nugaal, Middle Shabelle, Banadir, Bakool, Gedo, and Lower Juba were generally less likely to decide together with their partners. Urban residence increased the probability of self-decision-making (RRR = 1.166, 95% CI: 1.065–1.278, *p* < 0.001), while nomadic women were less likely to decide independently (RRR = 0.42, 95% CI: 0.373–0.473, *p* < 0.001). Wealth was significantly associated with autonomy, with women in the middle (RRR = 2.474, 95% CI: 2.211–2.768, *p* < 0.001), fourth (RRR = 2.528, 95% CI: 2.257–2.832, *p* < 0.001), and highest quintiles (RRR = 2.677, 95% CI: 2.364–3.03, *p* < 0.001) showing significantly higher relative risk ratios for self-decision-making compared to women in the lowest quintile; similarly, joint decision-making was higher among women in the middle (RRR = 1.316, 95% CI: 1.208–1.434, *p* < 0.01) and highest wealth quintiles (RRR = 1.907, 95% CI: 1.737–2.093, *p* < 0.001). Women whose husbands were unemployed were more likely to decide independently (RRR = 1.553, 95% CI: 1.438–1.678, *p* < 0.001) and slightly more likely to decide jointly (RRR = 1.065, 95% CI: 1.005–1.128, *p* < 0.05). Maternal education positively influenced autonomy: women with primary (self: RRR = 1.273, 95% CI: 1.135–1.428, *p* < 0.001; joint: RRR = 1.221, 95% CI: 1.116–1.336, *p* < 0.01), secondary (joint: RRR = 1.387, 95% CI: 1.177–1.635, *p* < 0.01), or higher education (self: RRR = 1.677, 95% CI: 1.046–2.69, *p* < 0.01; joint: RRR = 2.069, 95% CI: 1.442–2.967, *p* < 0.01) were more likely to make decisions themselves or jointly compared to women with no formal education. Distance to health facilities significantly affected autonomy, with women reporting a major problem being more likely to make decisions independently (RRR = 1.132, 95% CI: 1.052–1.219, *p* < 0.001) and jointly (RRR = 1.392, 95% CI: 1.317–1.471, *p* < 0.01). Age at first marriage also influenced decision-making, as women marrying at 20–24 (self: RRR = 1.297, 95% CI: 1.193–1.411, *p* < 0.001; joint: RRR = 1.083, 95% CI: 1.013–1.158, *p* < 0.01) and 25–30 years (self: RRR = 1.229, 95% CI: 1.048–1.44, *p* < 0.01; joint: RRR = 1.255, 95% CI: 1.104–1.427, *p* < 0.001) were more likely to participate in decision-making than those marrying before 20 years. Other household factors, including age of household head, sex of household head, and media exposure, also had significant associations with women’s autonomy. Overall, these findings indicate that socio-demographic, economic, educational, reproductive, and accessibility factors collectively shape women’s autonomy in healthcare decision-making in Somalia.

**Table 3:** Multivariable multinomial logistic regression analysis of associated factors on women’s autonomy in health care decision-making Among Reproductive-Age Women in Somalia

Variables	Categories	Women’s autonomy in health care decision making	
		Self	Together
		RRR (95% CI) *p*-V*	RRR (95% CI) *p*-V*
Age	15–19	Ref	-
	20–24	0.853(0.643, 1.132)	1.21(0.97, 1.511) *
	25–29	0.919(0.696, 1.215)	1.157(0.928, 1.443)
	30–34	1.175(0.885, 1.56)	1.403(1.12, 1.757) ***
	35–39	1.083(0.812, 1.444)	1.352(1.077, 1.699) ***
	40–44	1.446(1.077, 1.94) **	1.43(1.132, 1.808) ***
	45–49	1.933(1.424, 2.624) ***	1.611(1.259, 2.062) ***
Region	Awdal	Ref	-
	Woqooyi Galbeed	1.545(1.262, 1.891) ***	1.146(0.99, 1.326) *
	Togdheer	1.734(1.424, 2.112) ***	0.838(0.723, 0.972) **
	Sool	1.184(0.968, 1.448)	1.159(1.006, 1.334) **
	Sanaag	1.217(1, 1.483) *	0.696(0.602, 0.804) ***
	Bari	1.006(0.806, 1.255)	1.173(1.009, 1.364) **
	Nugaal	1.409(1.14, 1.741) ***	0.788(0.672, 0.925) ***
	Mudug	1.08(0.863, 1.351)	0.856(0.728, 1.006) *
	Galgaduud	1.244(0.994, 1.557) *	1.231(1.051, 1.441) **
	Hiraan	1.194(0.945, 1.51)	1.15(0.977, 1.355) *
	Middle Shabelle	1.065(0.854, 1.33)	0.789(0.672, 0.927) ***
	Banadir	1.074(0.879, 1.312)	0.854(0.736, 0.991) **
	Bay	1.406(1.059, 1.867) **	1.219(0.971, 1.53) *
	Bakool	0.457(0.362, 0.576) ***	0.555(0.477, 0.647) ***
	Gedo	0.207(0.156, 0.275) ***	0.263(0.221, 0.314) ***
	Lower Juba	0.373(0.293, 0.475) ***	0.407(0.346, 0.48) ***
Type of place of residence	Rural	Ref	-
	Urban	1.166(1.065, 1.278) ***	0.989(0.92, 1.063)
	Nomadic	0.42(0.373, 0.473) ***	0.63(0.578, 0.687) ***
Wealth index	Lowest	Ref	-
	Second	1.321(1.185, 1.474) ***	0.985(0.91, 1.067)
	Middle	2.474(2.211, 2.768) ***	1.316(1.208, 1.434) ***
	Fourth	2.528(2.257, 2.832) ***	1.294(1.185, 1.412) ***
	Highest	2.677(2.364, 3.03) ***	1.907(1.737, 2.093) ***
Husband employment	Yes	Ref	-
	No	1.553(1.438, 1.678) ***	1.065(1.005, 1.128) **
Husband’s educational status	No	Ref	-
	Yes	0.933(0.835, 1.044)	1.171(1.079, 1.27) ***
Maternal employment	Yes	Ref	-
	No	0.254(0.191, 0.337) ***	0.473(0.365, 0.613) ***
Maternal educational status	No Education	Ref	-
	Primary	1.273(1.135, 1.428) ***	1.221(1.116, 1.336) ***
	Secondary	1.09(0.86, 1.381)	1.387(1.177, 1.635) ***
	Higher	1.677(1.046, 2.69) **	2.069(1.442, 2.967) ***
Sex of household head	Male	Ref	-
	Female	1.039(0.966, 1.117)	1.111(1.051, 1.174)
Age of household head	15–24 years	Ref	-
	25–34 years	0.957(0.87, 1.053)	0.968(0.896, 1.045)
	35 and above	0.826(0.729, 0.937) ***	0.777(0.704, 0.858) ***
Family size	Less than four members	Ref	-
	Four members or more	1.053(0.97, 1.144)	1.032(0.97, 1.098)
Parity	Primipara	Ref	-
	Multipara	0.971(0.769, 1.226)	0.981(0.819, 1.176)
	grand multipara	0.908(0.713, 1.157)	1.025(0.849, 1.237)
Age at First Marriage	< 20	Ref	-
	20–24	1.297(1.193, 1.411) ***	1.083(1.013, 1.158) **
	25–30	1.229(1.048, 1.44) **	1.255(1.104, 1.427) ***
	30 and above	1.34(0.8, 2.245)	0.798(0.487, 1.307)
Distance to health facility	Not a big problem	Ref	-
	Big problem	1.132(1.052, 1.219) ***	1.392(1.317, 1.471) ***
media exposure	No	Ref	-
	Yes	1(0.996, 1.166) *	0.942(0.885, 1) **

*** *p*<0.01, ** *p*<0.05, * *p*<0.1 *Ref* reference category, *RRR* relative risk ratios, *CI* confidence interval

## Discussion

This study examined women in health care decision-making and its associated factors among reproductive-age women in Somalia using national representative survey data. The findings indicate that women’s autonomy remains limited and is strongly influenced by socio-demographic, economic, educational, reproductive, and contextual factors.

Age was significantly associated with decision-making, with older women more likely to make independent or joint decisions compared with adolescents. This suggests that autonomy increases with age, life experience, marital duration, and accumulated social capital. Similar findings have been reported in previous studies [1, 6, 7], where older age consistently predicted greater autonomy in health-related decision-making, limited negotiation power, and dependence on husbands or elders, which constrain their participation in health decisions. Marked regional disparities in autonomy were observed, with women in Marodijeh and Sahil regions exhibiting significantly higher levels of autonomous decision-making, while those in Togdheer, Sool, Gedo, and Lower Juba demonstrated lower autonomy. These regional variations may reflect differences in education level, economic development, cultural norms, security conditions, and availability of health services. In addition, urban women were more likely to make independent health care decisions compared with rural and nomadic women, which is Similar to this study’s finding. Other related studies [1, 8–10]. Indicating that urban residence facilitates better access to health facilities, information, and social support networks that promote female empowerment. Nomadic populations, in contrast, often experience geographical isolation, limited-service availability, and deeply rooted patriarchal traditions, which may further restrict women’s autonomy.

Socioeconomic status emerged as a significant predictor of autonomy. Women from middle to highest wealth quintiles were significantly more likely to participate in decision-making compared with the poorest households. Economic security may enhance women’s bargaining power, reduce financial dependence, and allow greater control over household resources, strengthening autonomy. This aligns with studies in Bangladesh and Nigeria [7, 9], which consistently demonstrate a strong positive association between household wealth and women’s decision-making power. Similarly, maternal employment was positively associated with autonomy, highlighting the role of economic participation in enhancing women’s confidence, independence, and negotiating capacity within households. Maternal education also showed a strong and consistent association with autonomy, particularly among women with secondary and higher education.

Education showed a strong association with autonomy, particularly among women with secondary or higher education. Education likely improves health literacy, awareness of rights, communication skills, and critical thinking, empowering women to engage actively in decisions concerning their health [9, 11–14], where education has been identified as a key driver of women’s empowerment and utilization of maternal health services. Conversely, women whose husbands were unemployed demonstrated higher levels of independent decision-making, which may reflect shifts in household power dynamics that compel women to assume greater responsibility for health-related decisions when male economic contribution is limited. Women’s autonomy was further affected by household and reproductive features. Perhaps as a result of conventional authority systems that give senior family members priority in home administration, women living in households led by elderly people were less likely to make independent choices. Women who married later in life showed more engagement in health-care choices, but those who married young were also linked to less autonomy.

Early marriage often limits women’s autonomy by restricting their access to education, fostering reliance, and solidifying patriarchal dominance. These results are in line with data from sub-Saharan Africa and South Asia, where early marriage is closely associated with lower levels of autonomy and worse maternal health outcomes. Access-related factors were also positively associated. Women who viewed the distance to health facilities as a significant issue were more inclined to report independent or collaborative decision-making, potentially suggesting that women encountering access barriers must actively negotiate or determine whether to pursue care. Even though media exposure was linked to autonomy in the bivariate analysis, it didn’t stay important in the multivariable model. This means that its effect might be caused by education, wealth, or living in a city instead of being a separate factor [1, 6, 14, 15].

## Conclusion

This study provides evidence that women’s autonomy in health-care decision-making in Somalia is influenced by a complex interplay of socio-demographic, economic, educational, reproductive, and contextual factors. Despite gradual improvements, a substantial proportion of women remain unable to make health-care decisions independently or jointly. Greater autonomy was observed among older women, those with higher maternal education, residents of wealthier households and urban areas, and women who married later. Conversely, women from poorer households, nomadic communities, early marriages, and resource-limited regions exhibited lower autonomy.

These findings highlight that women’s autonomy is shaped not only by individual characteristics but also by household structures, community norms, and broader socioeconomic conditions. From a policy perspective, integrated multi-sectoral strategies are essential to enhance women’s decision-making power, which is crucial for improving maternal and reproductive health outcomes. Interventions should focus on expanding girls’ access to quality education beyond the primary level, creating economic opportunities for women, delaying early marriage, transforming harmful gender norms, and reducing geographic and financial barriers to health services through improved infrastructure, mobile clinics, and community-based outreach programs.

### Strengths and limitations

This study has several important strengths. First, it utilized nationally representative data from the 2020 Somalia Demographic and Health Survey (SDHS), which enhances the generalizability of the findings to the broader population of reproductive-age women in Somalia. Second, the study employed an appropriate multinomial logistic regression model, allowing for a comprehensive analysis of different levels of women’s autonomy in healthcare decision-making rather than treating autonomy as a binary outcome. Third, the analysis accounted for the complex survey design by applying sampling weights, thereby improving the accuracy and validity of the estimates. Finally, the inclusion of a wide range of socio-demographic, economic, and reproductive variables provides a more holistic understanding of the factors influencing women’s autonomy in the Somali context.

Despite these strengths, several limitations should be acknowledged. First, the cross-sectional nature of the data limits the ability to establish causal relationships between the identified factors and women’s autonomy in healthcare decision-making. Second, the use of self-reported data may introduce recall bias and social desirability bias, particularly for sensitive issues related to household decision-making dynamics. Third, the Demographic and Health Survey (DHS) dataset does not capture important contextual factors such as cultural norms, intra-household power relations, and community-level influences, which may result in residual confounding. Fourth, some regions were excluded from the survey due to security constraints, which may affect full national representativeness. Finally, the analysis is based on data from a single survey year (2020), limiting the ability to assess trends or changes in women’s autonomy over time.

### Policy implications

The findings of this study have important policy implications for improving women’s autonomy in healthcare decision-making in Somalia. First, enhancing female education should be prioritized, particularly beyond the primary level, as education consistently emerged as a key factor associated with increased autonomy. Policies aimed at reducing school dropout rates and promoting girls’ secondary and higher education may substantially improve decision-making capacity.

Second, economic empowerment programs targeting women, including income-generating activities and employment opportunities, are essential to strengthen women’s bargaining power within households. Strengthening women’s financial independence may enhance their participation in healthcare decisions.

Third, targeted interventions are needed for rural and nomadic populations, where autonomy was significantly lower. Expanding access to healthcare services through mobile clinics, outreach programs, and improved infrastructure may reduce geographical barriers and support women’s engagement in decision-making.

Fourth, delaying early marriage through policy enforcement and community-based interventions may improve women’s autonomy, as later age at first marriage was associated with greater participation in decision-making.

Finally, multi-sectoral approaches involving health, education, and social protection sectors are required to address the structural determinants of gender inequality and promote women’s empowerment in Somalia.

## Data Availability

The Somalia Demographic and Health Survey (SDHS) 2020 datasets used in this study are publicly accessible and can be obtained through an online request at [https://microdata.nbs.gov.so/index.php/catalog/50], specifying the purpose of the research. The datasets are publicly available upon request from the Somalia National Bureau of Statistics.

## Abbreviations

DHS: Demographic and Health Survey
SDHS: Somalia Demographic and Health Survey
BDHS: Bangladesh Demographic and Health survey
CI: Confidence Interval
LMICs: Low- and Middle-Income countries
MMR: maternal Mortality Ratio
SSU: Secondary sampling Unit
